# Inflammatory Cytokine Expression Is Associated with Chikungunya Virus Resolution and Symptom Severity

**DOI:** 10.1371/journal.pntd.0001279

**Published:** 2011-08-16

**Authors:** Alyson A. Kelvin, David Banner, Giuliano Silvi, Maria Luisa Moro, Nadir Spataro, Paolo Gaibani, Francesca Cavrini, Anna Pierro, Giada Rossini, Mark J. Cameron, Jesus F. Bermejo-Martin, Stéphane G. Paquette, Luoling Xu, Ali Danesh, Amber Farooqui, Ilaria Borghetto, David J. Kelvin, Vittorio Sambri, Salvatore Rubino

**Affiliations:** 1 Sardinia Research and Development (SaRD), University di Sassari, Sassari, Italy; 2 Immune Diagnostics and Research (IDR), Toronto Medical Discovery Tower (TDMT), Toronto, Ontario, Canada; 3 Experimental Therapeutics, University Health Network (UHN), Toronto, Ontario, Canada; 4 Dipartimento di Sanità Pubblica, Azienda Usl di Ravenna, Ferrara, Italy; 5 Agenzia Sanitaria e Sociale Regione Emilia-Romagna, Bologna, Italy; 6 Department of Hematology and Oncology, Alma Mater Studiorum - University of Bologna, Bologna, Italy; 7 National Centre of Influenza, Hospital Clínico Universitario de Valladolid, Valladolid, Spain; 8 Unidad de Investigación en Infección e Inmunidad - Microbiology Service, Hospital Clínico Universitario de Valladolid - Instituto de Estudios de Ciencias de la Salud de Castilla y León (IECSCYL), Valladolid, Spain; 9 Faculty of Medicine, Institute of Medical Science (IMS), University of Toronto, Toronto, Ontario, Canada; 10 Dipartimento di Scienze Biomediche, University di Sassari, Sassari, Italy; 11 PhD School in Biomolecular and Biotechnology Sciences, University of Sassari, Sassari, Italy; Centers for Disease Control and Prevention, United States of America

## Abstract

The Chikungunya virus infection zones have now quickly spread from Africa to parts of Asia, North America and Europe. Originally thought to trigger a disease of only mild symptoms, recently Chikungunya virus caused large-scale fatalities and widespread economic loss that was linked to recent virus genetic mutation and evolution. Due to the paucity of information on Chikungunya immunological progression, we investigated the serum levels of 13 cytokines/chemokines during the acute phase of Chikungunya disease and 6- and 12-month post-infection follow-up from patients of the Italian outbreak. We found that CXCL9/MIG, CCL2/MCP-1, IL-6 and CXCL10/IP-10 were significantly raised in the acute phase compared to follow-up samples. Furthermore, IL-1β, TNF-α, Il-12, IL-10, IFN-γ and IL-5 had low initial acute phase levels that significantly increased at later time points. Analysis of symptom severity showed association with CXCL9/MIG, CXCL10/IP-10 and IgG levels. These data give insight into Chikungunya disease establishment and subsequent convalescence, which is imperative to the treatment and containment of this quickly evolving and frequently re-emerging disease.

## Author Summary

Chikungunya virus (CHIKV) is transmitted by mosquitoes and causes a human disease clinically characterized by sudden appearance of high fever, rash, headache, nausea, and severe joint pain (the defining symptom). Chikungunya was identified in Africa and the word *Chikungunya* means *that which bends up*, describing the bent posture of CHIKV patients while in severe pain. CHIKV, a current problem in Africa, Indian Ocean region, and Southeast Asia, is now spreading to temperate regions of North America, France and Italy. Presently, the immune response for CHIKV infection remains largely uninvestigated and no treatment is available. We investigated cytokine profiles at diagnosis and follow-up of CHIKV infected patients during the Italian 2007 outbreak and associated cytokine levels with antibody level and symptom severity. Cytokines, important immune mediators, are often drug targets. Since CHIKV symptoms can persist for months or years following infection it is important to investigate possible drug targets to alleviate discomfort. We found cytokine profiles that describe the initial infection and recovery phase. We determined the cytokines CXCL9/MIG and CXCL10/IP-10 as well as antibody levels were associated with symptom severity. These results reflect previously unreported cytokine profiles which may be important for the development of future therapeutics for CHIKV outbreaks.

## Introduction

The Chikungunya virus (CHIKV), an arthropod-borne virus (arbovirus), is a single-stranded positive-sense RNA virus with three genotypes. The virus is of the *Alphavirus* genus in the *Togaviridae* family [1], [2]. CHIKV has been shown to infect and be transmitted by *Ae. aegyptii* and *Ae. albopictus* mosquitoes. It was identified in East Africa in the early 1950s and since then has caused epidemics in continental Africa, the Indian Ocean region, and countries of Southeast Asia such as India, where since 2006 suspected cases have been estimated to be 1.39 million, and Singapore [3]–[6]. The only reported outbreak outside these areas was in Italy in the Emilia Romagna region in 2007. Small non-epidemic imported cases have been reported in other regions such as North America, France and Japan, which were caused by travelers returning from affected areas [7]–[9].

The epidemic occurring on La Reunion Island in the Indian Ocean remains the most devastating of all CHIKV outbreaks where over one-third of the population was affected [10]. During this outbreak, the CHIKV acquired a genetic mutation allowing the new vector *Ae. albopictus* mosquito to carry the virus where previously CHIKV only circulated in *Ae. aegyptii* mosquitoes [10], [11]. The *Ae. albopictus* differs in susceptibility to various genetically different isolates of the virus compared to the *Ae. aegyptii* [12]. CHIKV is now of global health concern since expansion of mosquito vectors has created potential for the Chikungunya virus to spread to temperate areas as *Ae. albopitcus* inhabits regions in North America and Europe [2], [13].

CHIKV infection is clinically characterized by the sudden appearance of high fever, rash, headache, nausea, vomiting, myalgia and arthalgia or severe joint pain. Severe joint pain is the defining symptom of CHIKV disease [11]. The word *Chikungunya* originated from the Tanzanian and Mozambique region of Africa meaning *that which bends up*. A bent posture is often taken by those in severe joint pain after being infected with CHIKV. CHIKV symptoms start 4 to 7 days after exposure and most resolve within the acute phase of the disease. Although the acute phase lasts approximately 2 weeks, joint pain can persist for months or years following initial infection [1], [14], [15]. Minimal research has been done investigating the immune response following CHIKV infection. Currently, there is no CHIKV specific therapeutic available.

The Italian outbreak of CHIKV spread through communities surrounding the city of Ravenna during August to October 2007 and also involved the major Italian city of Bologna [15], [16]. A recorded 254 people were identified to be infected through the *Ae. albopictus* mosquito which has inhabited the Emilia Romagna region since 1990 [14], [17], [18]. The virus brought to the Emilia Romagna region by a traveler returning from a CHIKV affected country was of the Central/East African genotype and matched most closely (100% amino acid identity) with the IND-06 virus isolated from the Reunion Island outbreak [14], [17]. The amino acid identity confirmed that this virus included a substitution mutation in the E1 envelope protein (E1-A226V) [19] which is important for viral entry into host cells. This mutation was acquired during the 2005-2006 Indian Ocean CHIKV outbreak and increased the virus's infectivity to the *Ae. albopictus* mosquito [20].

Cytokines are important immune mediators that conduct immune responses. Recently, cytokine profiles have been investigated in CHIKV infected humans by two groups [21], [22]. Ng and colleagues established cytokine profiles from 10 CHIKV patients that were infected during the Singapore 2007 CHIKV outbreak [22]. Although this study reported that IL-1β, IL-6 and RANTES were correlated with severe acute phase CHIKV disease, cytokine profiles were not determined for the progression and convalescence of the disease. Here we investigated cytokine profiles during the acute phase and 6- and 12-month follow-up of CHIKV infected patients of the Italian 2007 outbreak. Since CHIKV disease can have severe acute phase symptoms and be followed by persistent symptoms in the convalescence phase it was important to investigate the immune response responsible for these maladies. Furthermore, the Italian CHIKV included the A226V mutation and the Singapore virus did not. Furthermore, we analysed the relationship between cytokine levels and patient severity, and IgG levels linking high CXCL9, CXCL10 and IgG levels with disease severity. Therefore, the results presented here are virus specific and reflect previously unreported cytokine profiles which may be important for the development of future therapeutics for CHIKV outbreaks.

## Materials and Methods

### Ethics statement

Patients all gave written consent to the participation in scientific studies. Permission to perform scientific studies was given by Comitato Etico di Area Vasta Romagna Et IRSTof the Servizio Sanitario Regionale Emilia-Romagna, Italy.

### Objectives

Since the immune response during CHIKV disease has not been extensively investigated, our objectives were to create a clear clinical picture of CHIKV disease at the acute phase and during convalescence at 6- and 12-month follow-up by cytokine profiling. To achieve this objective, we investigated the cytokine profiles from patients at the acute phase and at 6- and 12-month follow-up.

### Participants

Included patients were from the region of Emilia-Romagna in north-east Italy suspected to be infected with CHIKV since they showed symptoms such as myalgia, severe back and joint pain, headache, and skin rash. Collaboration with the regional microbiology reference laboratory of Bologna University and the Department of Infectious and Parasitic Diseases of the National Institute of Health in Rome was initiated and identified the patients as having CHIKV. The clinical criteria was described as acute onset of fever (>38.5°C) and severe arthralgia not explained by other medical conditions. CHIKV infection was confirmed by one or more of the acute phase tests: virus isolation, reverse transcriptase-PCR (RT-PCR) positive for CHIKV *nsp1* gene, seroconversion to virus-specific serum antibodies collected at least 1 to 3 weeks apart, or presence of virus-specific IgM antibodies in a single serum sample collected [15]. Acute CHIKV patient samples were determined to be in the viral stage if the sample was PCR positive for CHIKV (7 patients), in the IgM antibody initiation stage if the sample was PCR negative, IgM positive, IgG negative (6 patients) or in the seroconversion stage (22 patients) if the sample was PCR negative, IgM positive and IgG positive. The samples were considered to be high IgG if the IgG level was greater than 6400 (6 months) (31 patients high out of 50) or greater than 3200 (12 months) (20 patients out of 50). IgG levels below or equal to these thresholds were considered low IgG. CHIKV patients were considered to be non-symptomatic (15 patients out of 50 at the 6-month follow-up; 34 patients out of 50 at the 12 month follow-up), have mild symptoms (21 patients out of 50 a the 6 month follow-up; 14 patients out of 50 at the 12 month follow-up), or have severe symptoms (14 patients out of 50 at the 6 month follow-up; 2 patients out of 50 at the 12 month follow-up) based on their responses to a questionnaire at the time of sampling which was based on: articular pain, muscle pain, mono-arthritis, oligo-arthritis, symmetric polyarthritis, asymmetric polyarthritis, tenosynovitis, arthralgia and fibromyalgia. Control samples were collected from 10 healthy volunteers screened for symptoms of viral infection.

### Patient sample collection

Blood samples were collected from consenting CHIKV positive patients at the time of diagnosis. Viral infection was determined as described above. Two follow-up samples were then collected from each patient at the 6-month evaluation and the 12-month evaluation. After sampling, serum was extracted and immediately frozen at −80°C until serum analysis.

### CBA

Serum samples were analyzed for cytokine levels using BD™ Cytometric Bead Array (CBA) Human Chemokine Kit, Human Inflammatory Cytokine Kit, and Human Th1/Th2 Cytokine Kit (BD Biosciences) according to the manufacturer's instructions for a total of 13 cytokines. Capture Beads were added to the serum sample followed by the PE detection reagent. The samples were then incubated for 3 hours at room temperature and washed with the assay Wash Buffer and resuspended again in Wash Buffer for analysis on the Flow Cytometer. CBAs were then run on a BD FACSCalibur Flow Cytometer.

### Statistical analysis

CBA data was analysed using SPSS statistical software. Box-whisker plots were created from the CBA FACS raw data. Six-month and 12-month samples were compared to the acute samples using the non-parametric two-tailed Wilcoxon signed-rank test for related samples to determine statistical significance. Each acute phase, 6-month and 12-month cytokine sample sets were statistically compared to healthy control CBA data using the non-parametric two-tailed Mann Whitney Test for unrelated samples.

## Results

### CHIKV resolution is associated with differential cytokine programs of increasing and decreasing trends

CHIKV causes a disease of crippling joint pain that has affected most of Asia and has demonstrated the capability to spread to non-tropical areas such as Europe and parts of North America [1]. Cytokines are inflammatory mediators and their balance is often associated with inflammatory disease [23]. Previously, the cytokine profiles of acute phase CHIKV patients have been examined [22]. Here we profiled cytokine levels in acute phase and 6- and 12-month follow-up CHIKV patient serum samples to determine a cytokine signature that may correlate with acute symptoms, following persistent joint pain and/or disease resolution. Blood samples were collected from 50 patients suffering from CHIKV infections during the 2007 Italian outbreak. Serum separated from whole blood was analyzed by cytokine bead analysis (CBA) for 13 cytokines with the intention of determining a cytokine profile during CHIKV acute phase and disease convalescence. Three cytokine profiles emerged from our data: decreasing, increasing and no-trend.

**Figure 1:**
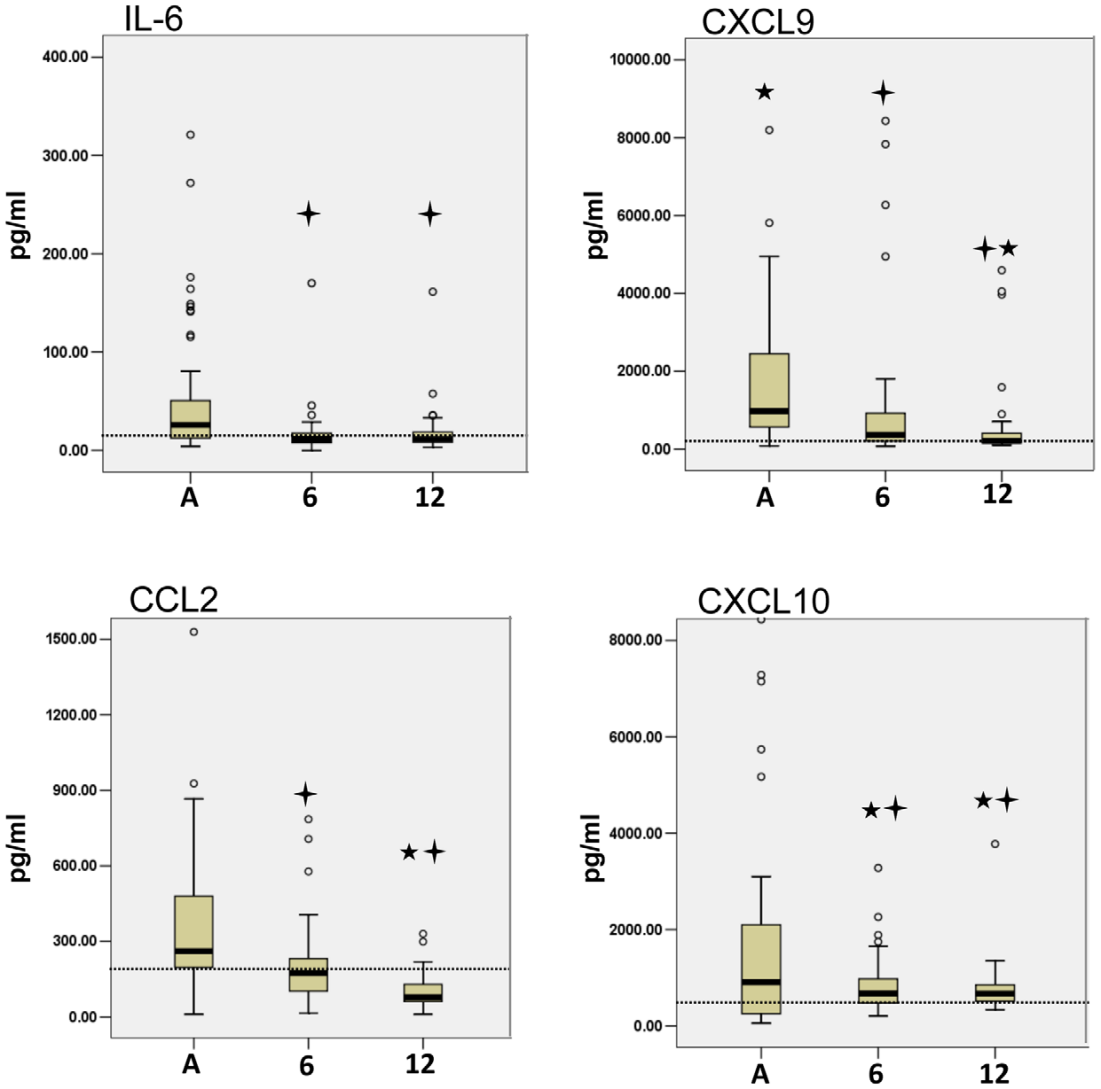
Acute phase CHIKV disease was associated with high levels of IL-6, CXCL9, CCL2, CXCL10. Cytokine Bead Array analysis of CHIKV patient serum samples showed high levels of IL-6, CXCL9, CCL2 and CXCL-10 are associated with acute disease phase and decreased with patient convalescence. Six-month and one-year cytokine levels were analysed for statistical significance using the Wilcoxon test for Significance by comparing with acute levels. All samples were also analyzed for significance against healthy controls by the Mann-Whitney U test. The cross symbol indicates a p-value less than 0.05 for 6- and 12-month groups compared to acute values and star symbol indicates a p-value less than 0.05 for acute, 6- and 12-month groups compared to control values. The dotted line indicates the median of healthy control cytokine levels. Acute (A), 6-month follow-up (6), and 12-month follow-up (12).

The first trend showed cytokine levels significantly higher in the acute samples compared to the follow-up time points revealing a decreasing pattern as patients left the acute phase. CXCL9/MIG (CXCL9), IL-6, CCL2/MCP-1(CXCL2) and CXCL10/IP-10 (CXCL10) cytokines had significantly decreased at both 6-month and 12-month follow-ups (Figure 1). Interestingly, some patients had extremely high levels in the acute phase; CXCL9 and CXCL10 levels decreased 1000 fold to 10,000 fold during convalescence. Furthermore, the decreasing trends for IL-6, CXCL9, and CXCL10 reached similar levels as those of the community control levels (shown by dotted line). CCL2 levels decreased significantly lower than the control levels by 12 months. Taken together, this data demonstrated that CXCL9, IL-6, CCL2 and CXCL10 were initially increased with acute CHIKV infection and decreased over time.

**Figure 2:**
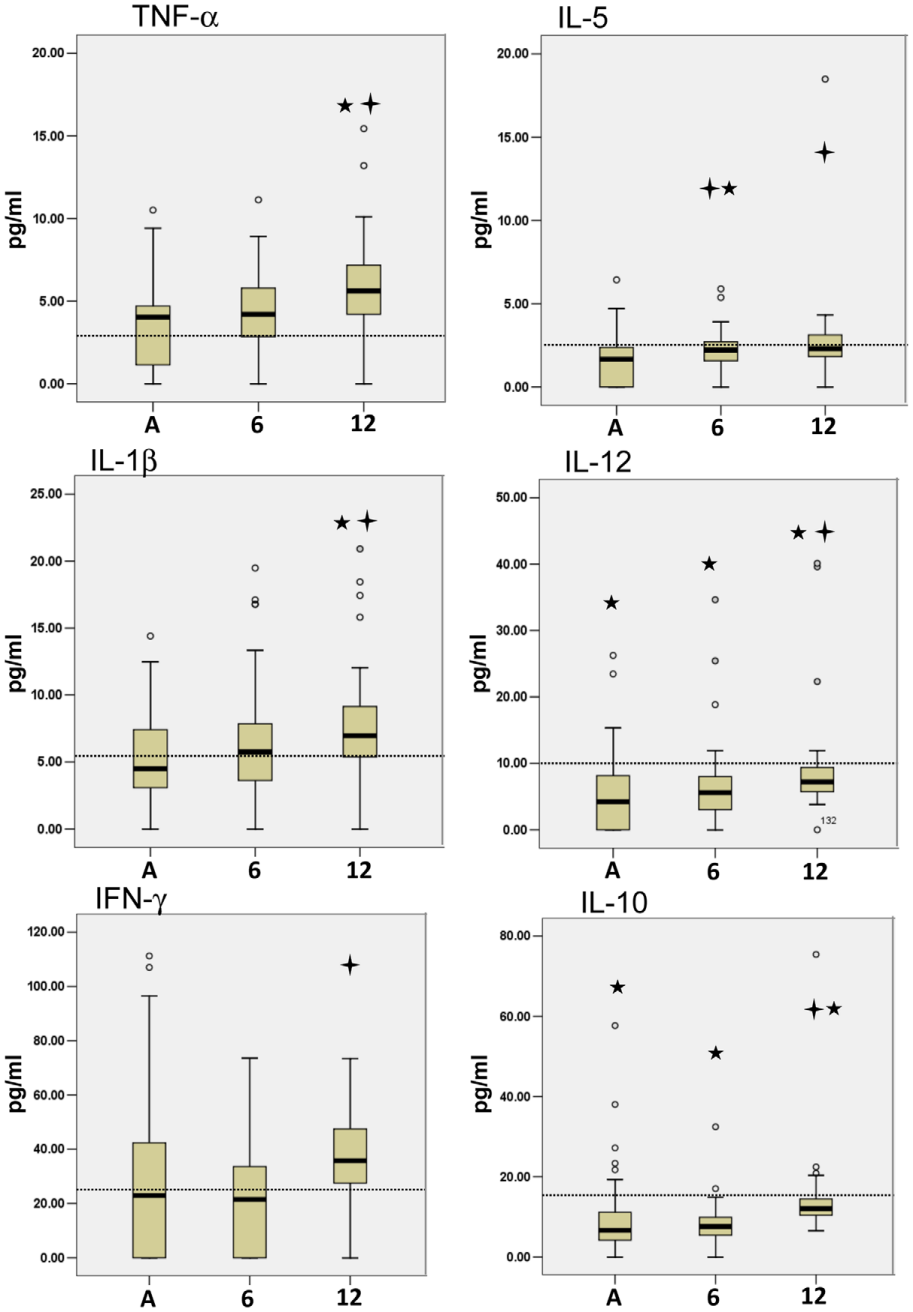
CHIKV patient convalescence was associated with increasing levels of TNF-α, IL-5, IL-1β, IL-12, IFN-γ and IL-10. Cytokine Bead Array analysis of CHIKV patient serum samples showed that following the acute phase of CHIKV disease patients had increasing levels of TNF-α, IL-5, IL-1β, IL-12, IFN-γ and IL-10. Six-month and one-year cytokine levels were analysed for statistical significance using the Wilcoxon test for Significance by comparing with acute phase values. As well, samples were analyzed for significance against healthy controls by the Mann-Whitney U test. The cross symbol indicates a p-value (Wilcoxon test) less than 0.05 for 6- and 12-month groups compared to acute values and star symbol indicates a p-value (Mann-Whitney U test) less than 0.05 acute, 6- and 12-month groups compared to control values. The dotted line indicates the median of healthy control cytokine levels. Acute (A), 6-month follow-up (6), and 12-month follow-up (12).

The second cytokine trend that emerged described cytokines that significantly increased following the acute phase. Cytokine profiles that were markedly lower in the acute phase and subsequently increased included IL-1β, TNF-α, IL-12, IL-5, IL-10 and IFN-γ (Figure 2). The cytokine increase was more gradual than the previous decreasing trend, where fold changes were generally closer to 2. Both the 6- and 12-month follow-up were statistically increased compared to acute values for IL-5 levels. IL-1β, TNF-α, IL-12, IL-10 and IFN-γ had significantly increased by 12 months. Even though the average for these cytokines had also risen by 6 months it was not significant. Furthermore, the increasing trends for TNF-α, IL-5, and IL-10 reached similar levels to the community control levels (shown by dotted line) and IFN-γ reached significantly higher than controls at 12 months. Interestingly, although IL-1β and IL-12 increased through the observed time, these cytokines stayed significantly lower than those of the controls. This data showed that cytokines IL-1β, TNF-α, IL-12, IL-5, IL-10 and IFN-γ increased in the convalescence phase of CHIKV infection.

**Figure 3:**
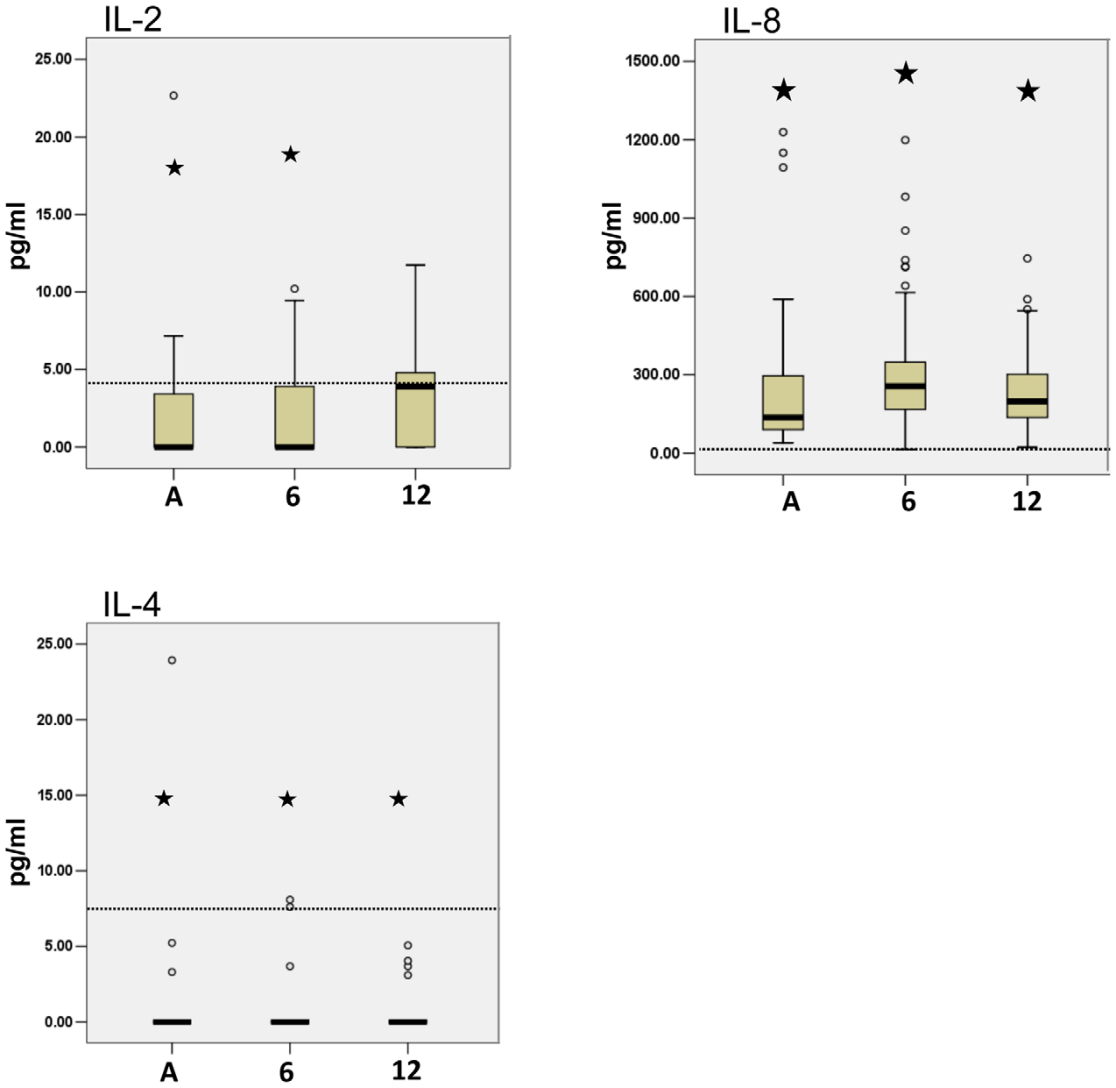
IL-2, IL-8 and IL-4 were not associated with acute or convalescence phase of CHIKV disease. Cytokine Bead Array analysis of CHIKV patient serum samples showed that IL-2, IL-8 and IL-4 were not associated with acute phase or convalescence of CHIKV disease in patients. Six-month and one-year cytokine levels were analysed for statistical significance using the Wilcoxon test for Significance by comparing with acute phase values. Samples were analyzed for significance against healthy controls by the Mann-Whitney U test. The star symbol indicates a p-value (Mann-Whitney U test) less than 0.05 acute, 6- and 12-month groups compared to control values. The dotted line indicates the median of healthy control cytokine levels. Acute (A), 6-month follow-up (6), and 12-month follow-up (12).

No significant change was seen for IL-2, IL-4, and IL-8 from the acute phase to the 12-month follow-up (Figure 3). Interestingly, IL-2 reached similar levels to those of the controls where IL-8 and IL-4 remained significantly raised and lowered, respectively.

### CXCL10, CXCL9, IL-6 and IL-10 are possible biomarkers of virus, IgM and IgG levels in CHIKV patient serum

**Figure 4:**
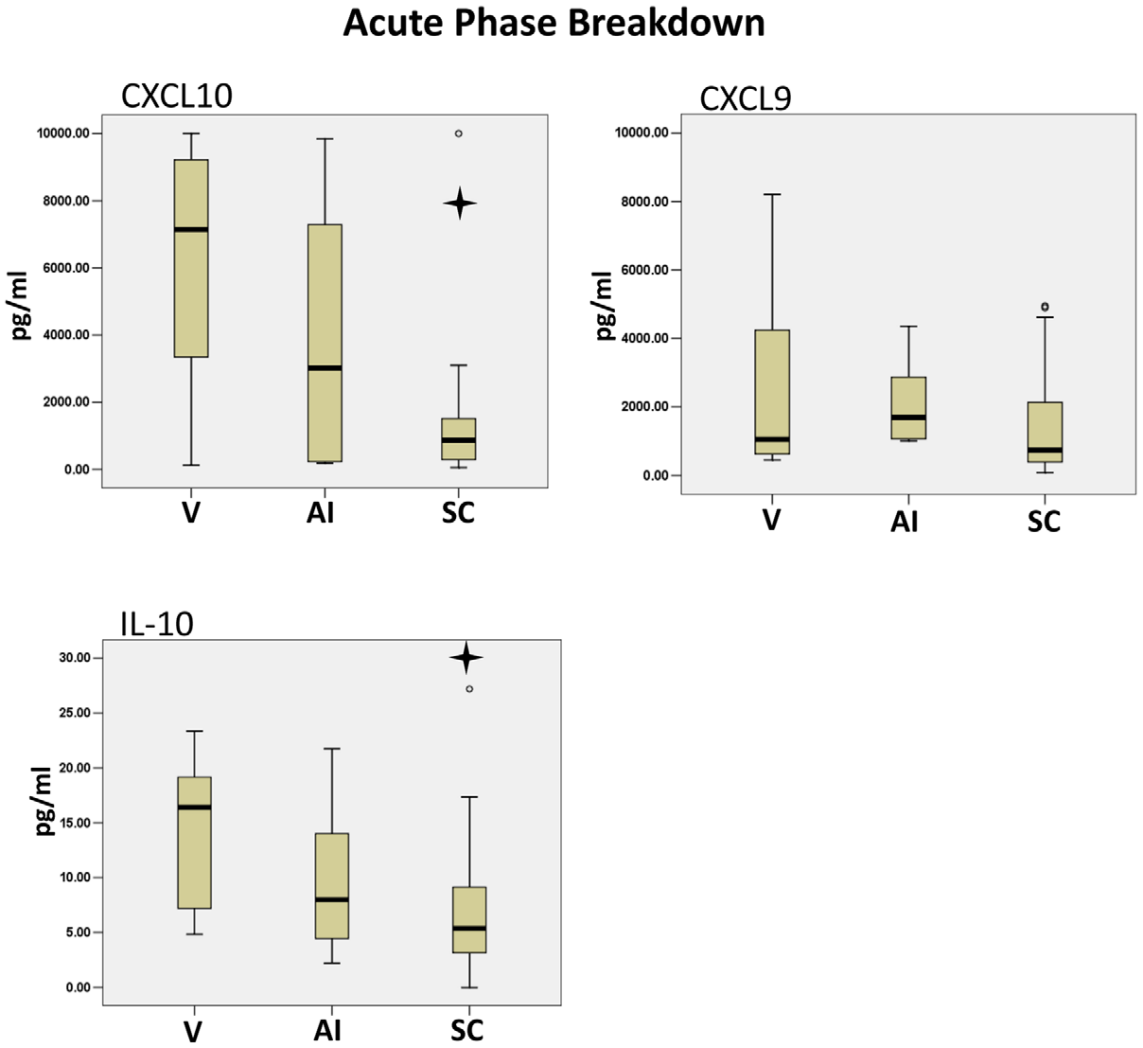
The stages of CHIKV Acute phase were marked by changes in CXCL10 and IL-10. Acute CHIKV patients were categorized into Viral stage (V), Antibody Initiation stage (AI) or Seroconversion stage (SC) according to the presence of CHIKV, IgM and IgG antibodies. Cytokine Bead Array analysis of the serum samples showed a significant decrease in CXCL10 and IL-10 from the Viral stage to the Seroconversion stage of the Acute phase. A Mann-Whitney U test was used to determine significance among the phases. The star symbol indicates a p-value less than 0.05 compared to the Viral phase.

Previously, we have shown that the acute phase of West Nile Virus (WNV) can be described in 3 stages [24]. Since the samples from the CHIKV patients were taken at various stages of the acute phase, we went on to determine if there were cytokines marking the viral (V), IgM antibody initiation (AI) or seroconversion (SC) stage. Acute CHIKV patient samples were determined to be PCR positive for CHIKV (viral stage), IgM positive, IgG negative (IgM antibody initiation stage) or IgM positive, IgG postive (seroconversion stage). Samples were put into Viral stage (V), Antibody Initiation stage (AI) or Seroconversion stage (SC) according to the presence of CHIKV, IgM and IgG antibodies. Cytokine Bead Array analysis of the serum samples showed a significant decrease in CXCL10 and IL-10 from the viral stage to the seroconversion stage of the acute phase (Figure 4). The median of CXCL10 in the viral stage was approximately 7000 pg/ml and dropped to less than 1000 pg/ml after seroconversion. Interestingly, the IL-10 median decreased by 3 fold from the viral stage to the seroconversion stage.

**Figure 5:**
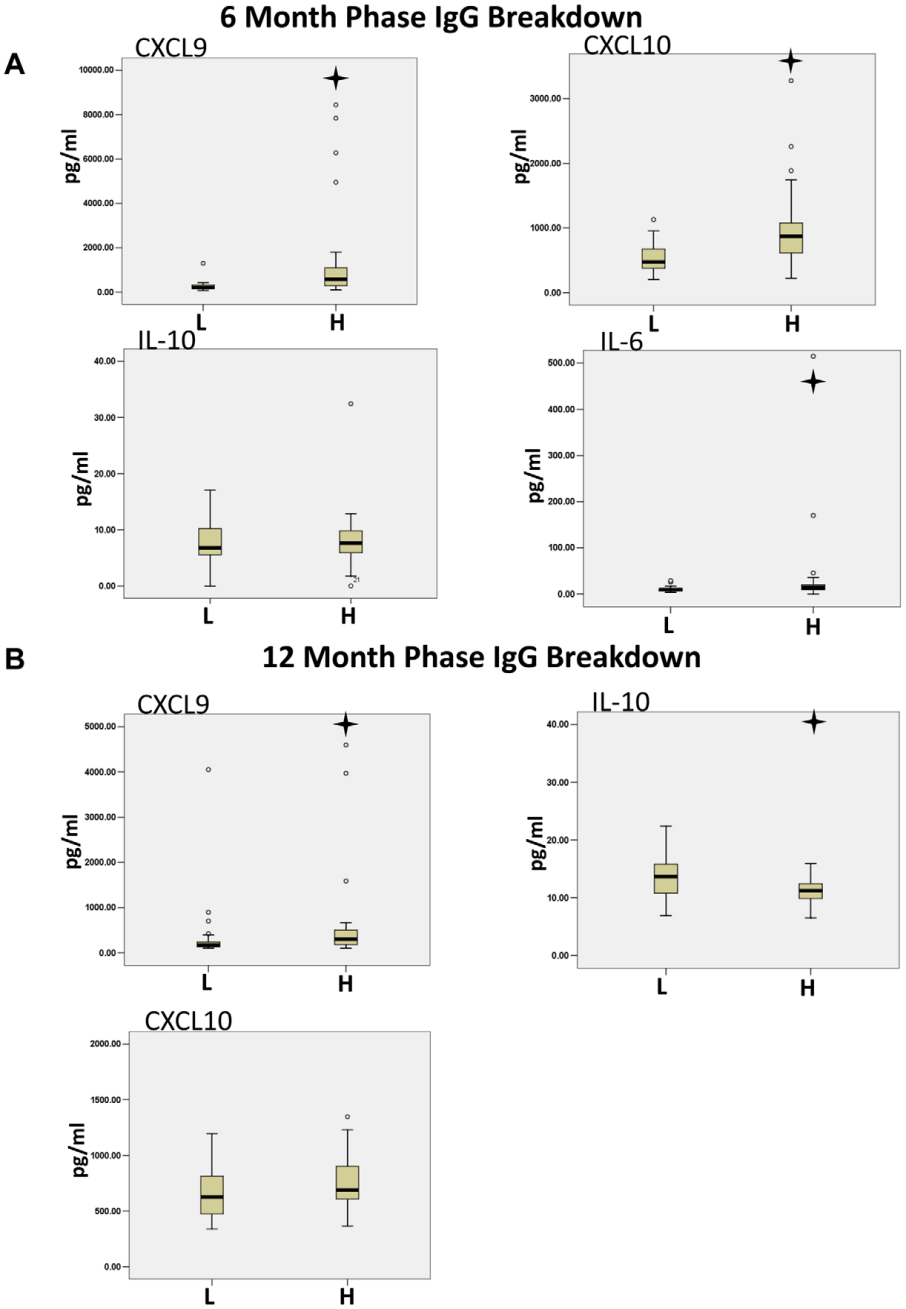
The stages of CHIKV 6- and 12-month follow-up phases are marked by differentials in CXCL10, CXCL9, IL-6 and CXCL9, IL-10 respectively. IgG levels in 6- and 12-month patient serum samples were determined by ELISA. The samples were then grouped by IgG level; a low IgG level group (L) and a high IgG level group (H). Cytokine Bead Array analysis of the serum samples showed a significant difference in CXCL9, CXCL10 and IL-6 between patients with high and low IgG levels at the 6-month time point. Twelve-month follow-up CHIKV patient samples showed a significant difference in CXCL9 and IL-10 between low (1) and high (2) IgG groups. A Mann-Whitney U test was used to determine significance among the IgG groups. The cross symbol indicates a p-value less than 0.05.

Next we sought to determine if the high levels of IgG in the follow-up phases were also significantly associated with cytokine levels compared to the cytokine levels of patients with low levels of IgG. The patients were put into an IgG high group (H) or an IgG low group (L) and the levels of each cytokine were statistically compared for each group using the Mann-Whitney U Test. In the 6-month follow-up phase CXCL9, CXCL10 and Il-6 were found to be statistically different between the high IgG group and the low IgG group (Figure 5A). High levels of all 3 cytokines were associated with high levels of IgG antibodies. IL-10 is also shown for comparison since it was statistically significant during the acute phase breakdown and the 12-month follow-up. Interestingly, in the 12-month follow-up phase, CXCL9 was found to be statistically higher in the IgG high group where IL-10 was significantly lower in the high IgG group (Figure 5B). CXCL10 is shown for comparison at 12 months although it was not significantly different between the IgG high and IgG low groups. In summary, the results suggested that CXCL9, CXCL10 and Il-6 were associated with IgG levels in the 6-month follow-up phase and CXCL9 and IL-10 in the 12-month follow-up phase.

### CXCL10, CXCL9 and IgG levels are possible biomarkers of CHIKV disease severity

**Figure 6:**
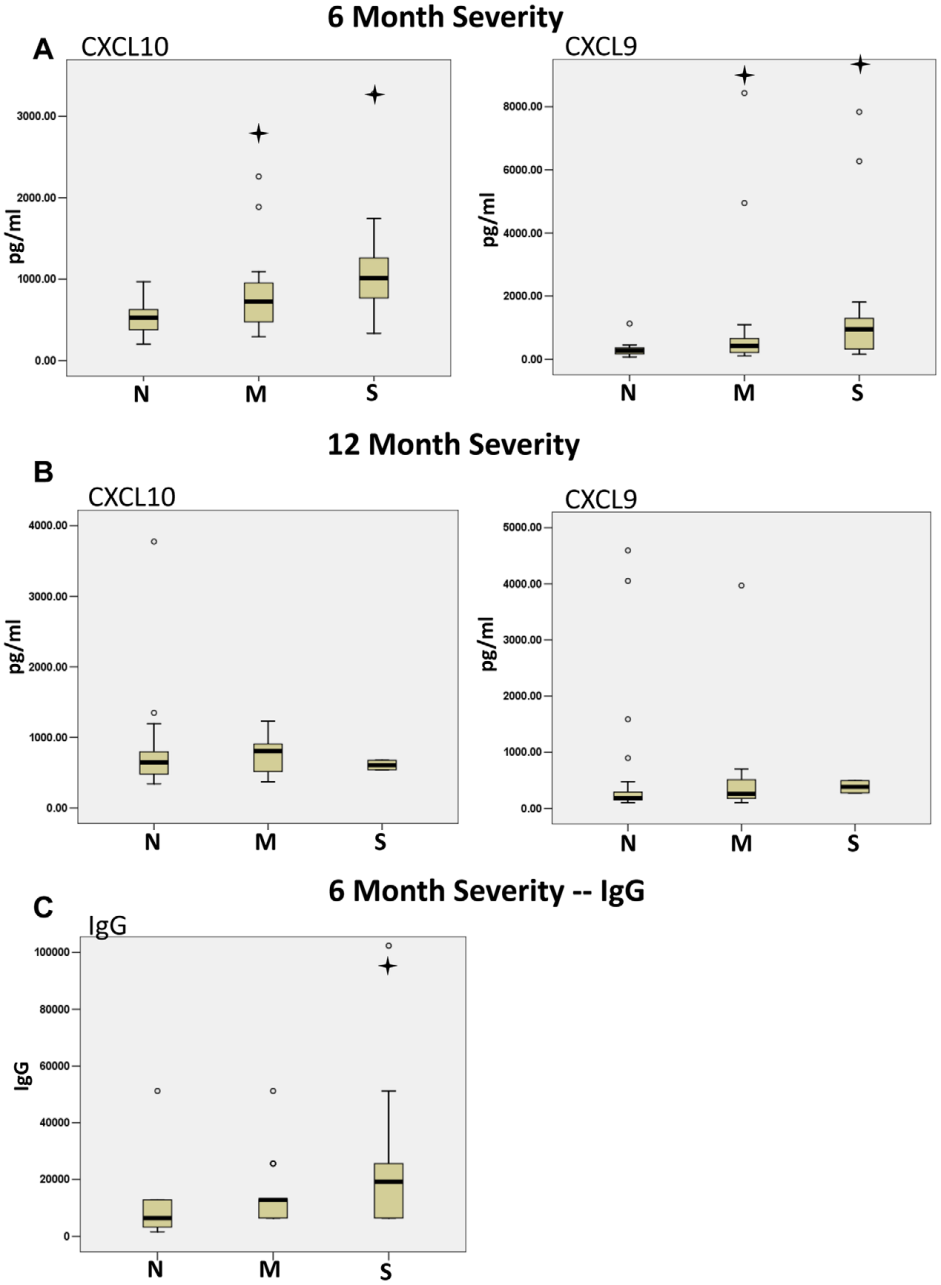
CHIKV disease severity is associated with high CXCL10, CXCL9 and IgG levels at the 6-month time point. CHIKV patients were determined to be nonsymptomatic (N), to have mild symptoms (M) or to have severe symptoms (S). The cytokine and IgG levels were then grouped by symptom level and a Mann-Whitney U test was used to determine significance among the severity groups. CXCL10, CXCL9 and IgG were found to be significantly increased in the patients with mild and severe symptoms at 6 months following initial infection. The cross symbol indicates a p-value less than 0.05.

IL-1β, IL-6 and RANTES were found to be associated with symptom severity of the Singapore 2007 CHIKV outbreak [22]. After determining the cytokine profiles of our Italian 2007 CHIKV patients during their disease resolution, we next sought to determine the association between symptom severity and cytokine levels. Patients were determined to be non-symptomatic (N), to have mild symptoms (M) or to have severe symptoms (S) depending on their responses to a questionnaire. The cytokine levels were then grouped by symptom level and a Mann-Whitney U test was used to determine significance among the severity groups for each cytokine. CXCL10 and CXCL9 were found to be significantly increased in the patients with mild and severe symptoms at 6 months following initial infection compared to the patients reporting no symptoms (Figure 6A). A 2 fold difference was seen between the medians of the non-symptomatic and severe patients for CXCL10 and a 5 fold difference for CXCL9. No statistical difference was seen for any of the 13 cytokines profiled for the 12-month follow-up. CXCL10 and CXCL9 at 12 months are shown for comparison (Figure 6B). Moreover, we analyzed the IgG levels at the 6-month follow-up in patients with no symptoms, mild symptoms, and severe symptoms. The results showed IgG levels were statistically increased with symptom severity (Figure 6C). Taken together, these results suggested CXCL10, CXCL9 and IgG to be possible markers of CHIKV severity during early phases of disease resolution.

## Discussion

CHIKV disease is a self-limiting disease caused by an *alphavirus* of the *Togaviridae* family. Although historically the virus only caused a disease of mild symptoms, a recent outbreak on La Reunion Island caused significant mortality due to genetic alterations [1], [20], [22], [25], [26]. Here we have investigated the immune response of an Italian population infected with the Indian Ocean genotype of CHIKV and have generated a cytokine signature for the initial infection to convalescence phase of CHIKV disease, the signature of viral and antibody production phases, and identified cytokines raised in patients with severe symptoms. We found that initial infection and the subsequent convalescence were described by a set of decreasing and increasing cytokines. Furthermore, we have shown that CXCL10 and IL-10 levels were associated with the viral stage of the acute phase and CXCL10 and CXCL9 with high IgG levels of the 6-month follow-up. As well, CXCL10 and CXCL9 were markers of symptom severity. Importantly, these identified signatures depict the immunological programs and may be key to the development of therapeutics for the frequently re-emerging CHIKV disease.

Our analysis has indentified 2 cytokine profiles that followed disease onset and continued with disease progression/convalescence. We found CXCL9, CXCL10, CCL2 and IL-6 levels were high in the acute phase and decreased as patients convalesced. Conversely, the trend for TNF-α, IL-1β, IL-2, IL-5 and IL-12 were low initially and increased as patients began to recover from acute illness. High levels of CXCL9, CXCL10, CCL2, and IL-6 in the acute phase possibly indicated an inflammatory program initiating adaptive T-cell immunity [27]. CXCL9 and CXCL10 are both chemokines induced by IFN-γ and are part of the chemokine program that regulates the migration of monocytes/macrophages, memory T cells and NK cells and are associated with the polarization of T cells [27]. IL-6, a pleiotropic cytokine, has a destructive role in rheumatoid arthritis (RA), contributing to joint inflammation as well as joint pain [28], [29]. The increased IL-6 levels in the acute phase of our study may be the initiating factor of the severe joint pain symptoms reported in CHIKV patients which mimics RA. Furthermore, IL-6 is important during acute phases of the disease by acting as an important immune mediator of fever activating muscle metabolism to increase core body temperature [28]. Since fever is a common symptom of acute CHIKV disease, it is highly probable that the high IL-6 levels were contributing to the acute fever and the IL-6 decreasing trend followed patient core body temperature as it returned to resting temperature in the follow-up.

A second host immune response profile was characterized by TNF-α, IL-1β, Il-10, Il-12, IFN-γ and IL-5, which increased from the acute phase into convalescence. Interestingly, TNF-α and IL-1β, which are known to co-induce the other's expression, are both involved in chronic inflammatory diseases such as RA, chronic hepatitis B and C infection [23], [29], [30]. Importantly, TNF-α and IL-1β are main contributors to joint pain, which is the major symptom of RA. An internal balance of TNF-α or IL-1β levels is imperative as mis-regulation of either has been shown to be a major proponent of chronic diseases (RA). Our data indicated that these cytokines increased significantly during patient convalescence above those of the control group, but were not statistically changed in patients reporting mild or severe symptoms. These data may imply that the increased levels of TNF-α and IL-1β in the convalescence phase were not a major contributor of chronic damage causing persistent severe joint pain symptoms during the Italian outbreak even though these cytokines have previously been found to play a destructive role in chronic inflammatory diseases. Furthermore, TNF-α immunomodulators have previously been used as a common treatment for RA and IL-1β immunomodulators are effective with other chronic diseases such as systemic-onset juvenile idiopathic arthritis and in adult onset Still's diseases [29], [31]. Taken together, these findings suggest TNF-α and IL-1β therapies would not be effective controlling the prolonged symptoms of CHIKV disease since the raised levels during convalescence were not associated with patient severity.

Previously, cytokine profiles have been analyzed from patients during an Asian outbreak of CHIKV [22]. Ng and colleagues, investigated 30 cytokines and growth factors from 10 acute phase CHIKV patients, determined that the levels of 8 cytokines, 2 chemokines and 3 growth factors were significantly raised in patients compared to those of the control group. In accordance with their data, IL-6, CXCL9 and CXCL10 were also increased in the acute phase of our patients as compared to control. Since the results from the previous study did not follow the patients during convalescence, our study added significant insight to the progression of CHIKV disease. We found that these three cytokine levels decreased as the patients convalesced as discussed above. Conversely, we found CCL2 also to be increased in the acute phase compared to that of the control group, which was unchanged in the Ng study. Furthermore, Ng *et al.* found IL-5 and IL-10 were significantly increased in the acute phase where our data indicated that IL-5 and IL-10 were initially low and below control levels and increased following the acute phase. These discrepancies can possibly be explained by the patient populations: the Ng study patients and our patients were from significantly varied genetic backgrounds (Asian and European, respectively). Therefore, differences in immune response may reflect variations in immunological genetic programs. As well, the virus that caused the two outbreaks also differed genetically. Even though the virus that caused the Singapore outbreak was the same genotype as the Italian virus, the virus that infected the Italian patients had the A226V mutation in the E1 gene which was acquired on La Reunion Island [19]. Although the impact of this mutation has been shown to increase vector infectivity [20], the mutation has not been investigated on the human or mammalian immune response.

Previously, we have identified 3 stages of the acute phase of WNV; a viral stage, antibody initiation stage, and seroconversion stage [24]. As the viral load decreased in the WNV patients, IgM antibody levels were initiated and followed by IgM conversion to IgG, thereby marking the 3 stages of the acute phase. From this work we were able to identify the stages of the CHIKV patients and compare their respective serum cytokine levels. We found CXCL10 and IL-10 levels decreased as patients progressed through the viral stage to seroconversion. Since CXCL10 is often correlated with viral load, this observation was not surprising [32]. High IL-10 levels in the viral stage may act in an effort to control the IFN-γ program [33] shown by high CXCL10 levels. Furthermore, plasma levels of CCL2, IL-6 as well as CXCL10 have all been correlated with viral loads in virus infected individuals [24], [34], [35]. It is possible that the decrease of CCL2 and IL-6 we observed subsequent to the acute phase [14] correlated with viral clearance, although not with antibody levels. Analysis of the cytokine/antibody response was carried on to the 6- and 12-month follow-up where we grouped the patients on their IgG levels. CXCL9, CXCL10 and IL-6 were raised in the patients with increased IgG levels at 6 months and CXCL9 at the 12-month follow-up. These proinflammatory cytokine associations with high IgG levels may represent the persistence of an active immune system.

CXCL9 and CXCL10 as well as high IgG levels were found to be biomarkers of severe CHIKV symptoms. Previously, CXCL10 has been associated with severe viral disease supporting a role for CXCL10 in severe CHIKV disease [34]–[37]. These studies did not find an association with CXCL9 and severity as seen in our CHIKV patients. Our findings suggest high CXCL10 and CXCL9 associated with severity to be a unique signature of CHIKV. Interestingly, not only are CXCL10 and CXCL9 expressed in RA and other arthritis related disease patients [38]–[41], but they have been shown to be biomarkers for RA symptoms, implying a similar mechanism for joint destruction in CHIKV disease [42]. Moreover, CXCL9 and CXCL10 may be contributors of persistent immune activation in CHIKV disease leading to chronic symptoms, which implies cytokine immunomodulation may significantly improve patient treatment and recovery. Importantly, CXCL10 also has prognostic value in the treatment of viral hepatitis where CXCL10 levels follow disease recovery [43] supporting our finding and proposes a prognostic role for CXCL10 in CHIKV. Furthermore, our data puts forth CXCL10 and CXCL9 as possible drug targets for treatment of CHIKV symptoms in the convalescence phase due to the association with severity; however, further investigation is needed on CXCL10 and CXCL9 efficacy. In addition, not only are the identified cytokines useful as possible drug targets but the cytokine signatures described can also be applied when testing newly developed CHIKV therapeutics. As CHIKV therapeutics are evaluated in the CHIKV disease model, cytokine profiles can be used as an output for determining the efficacy of the novel therapeutics.

The synovial mast cell remains an important component during RA joint destruction by the exocytosis of intracellular granules containing inflammatory mediators. Currently, mast cell activation through FcγRs by high levels of circulating IgG antibodies is hypothesized to contribute to the pathological destruction of synovium in RA [44]–[46]. In addition, antibody immune complex formation within the joint stabilizing inflammatory mediators, such as chemokines and complement proteins, is another possible facet of pathogenesis during RA [47], [48]. We found high concentrations of IgG to be associated with symptom severity in CHIKV patients. Similar IgG mediated mechanisms leading to synovium destruction and severe pain experienced by CHIKV patients are possible. Taken together with the roles of IgG in RA, our findings support the need for further investigation into the contribution of IgG levels and mast cells to CHIKV symptoms.

We have found that the cytokines investigated had one of two immunologically important profiles during CHIKV disease onset and convalescence; furthermore, CXCL10 and CXCL9 were makers of disease severity. By identifying the immune profiles, we have created a clearer clinical picture of CHIKV disease. Importantly, further investigation is needed to correlate these profiles with disease onset and progression to use the cytokine profiles as biomarkers for severity and symptom persistence. The data presented here suggest that with further investigation, immunomodulators may significantly enhance patient recovery.

## References

[1] Cavrini F, Gaibani P, Pierro AM, Rossini G, Landini MP (2009). Chikungunya: an emerging and spreading arthropod-borne viral disease.. J Infect Dev Ctries.

[2] Sudeep AB, Parashar D (2008). Chikungunya: an overview.. J Biosci.

[3] Demanou M, Antonio-Nkondjio C, Ngapana E, Rousset D, Paupy C (2010). Chikungunya outbreak in a rural area of Western Cameroon in 2006: A retrospective serological and entomological survey.. BMC Res Notes.

[4] Niyas KP, Abraham R, Unnikrishnan RN, Mathew T, Nair S (2010). Molecular characterization of Chikungunya virus isolates from clinical samples and adult Aedes albopictus mosquitoes emerged from larvae from Kerala, South India.. Virol J.

[5] Santhosh SR, Dash PK, Parida M, Khan M, Rao PV (2009). Appearance of E1: A226V mutant Chikungunya virus in Coastal Karnataka, India during 2008 outbreak.. Virol J.

[6] (2011). NVBDCP (2007).. Chikungunya fever situation in the country during 2006.

[7] Parola P, de L, X, Jourdan J, Rovery C, Vaillant V (2006). Novel chikungunya virus variant in travelers returning from Indian Ocean islands.. Emerg Infect Dis.

[8] Gibney KB, Fischer M, Prince HE, Kramer LD, St GK (2011). Chikungunya fever in the United States: a fifteen year review of cases.. Clin Infect Dis.

[9] Mizuno Y, Kato Y, Takeshita N, Ujiie M, Kobayashi T (2010). Clinical and radiological features of imported chikungunya fever in Japan: a study of six cases at the National Center for Global Health and Medicine.. J Infect Chemother.

[10] Schuffenecker I, Iteman I, Michault A, Murri S, Frangeul L (2006). Genome microevolution of chikungunya viruses causing the Indian Ocean outbreak.. PLoS Med.

[11] Thiboutot MM, Kannan S, Kawalekar OU, Shedlock DJ, Khan AS (2010). Chikungunya: a potentially emerging epidemic?. PLoS Negl Trop Dis.

[12] Vazeille M, Moutailler S, Coudrier D, Rousseaux C, Khun H (2007). Two Chikungunya isolates from the outbreak of La Reunion (Indian Ocean) exhibit different patterns of infection in the mosquito, *Aedes albopictus*.. PLoS One.

[13] De L, X, Leroy E, Charrel RN, Ttsetsarkin K, Higgs S (2008). Chikungunya virus adapts to tiger mosquito via evolutionary convergence: a sign of things to come?. Virol J.

[14] Sambri V, Cavrini F, Rossini G, Pierro A, Landini MP (2008). The 2007 epidemic outbreak of Chikungunya virus infection in the Romagna region of Italy: a new perspective for the possible diffusion of tropical diseases in temperate areas?. New Microbiol.

[15] Liumbruno GM, Calteri D, Petropulacos K, Mattivi A, Po C (2008). The Chikungunya epidemic in Italy and its repercussion on the blood system.. Blood Transfus.

[16] Seyler T, Rizzo C, Finarelli AC, Po C, Alessio P (2008). Autochthonous chikungunya virus transmission may have occurred in Bologna, Italy, during the summer 2007 outbreak.. Euro Surveill.

[17] Bonilauri P, Bellini R, Calzolari M, Angelini R, Venturi L (2008). Chikungunya virus in Aedes albopictus, Italy.. Emerg Infect Dis.

[18] Charrel RN, de L, X (2008). Chikungunya virus in north-eastern Italy: a consequence of seasonal synchronicity.. Euro Surveill.

[19] Bordi L, Carletti F, Castilletti C, Chiappini R, Sambri V (2008). Presence of the A226V mutation in autochthonous and imported Italian chikungunya virus strains.. Clin Infect Dis.

[20] Tsetsarkin KA, Vanlandingham DL, McGee CE, Higgs S (2007). A single mutation in chikungunya virus affects vector specificity and epidemic potential.. PLoS Pathog.

[21] Chirathaworn C, Rianthavorn P, Wuttirattanakowit N, Poovorawan Y (2010). Serum IL-18 and IL-18BP levels in patients with Chikungunya virus infection.. Viral Immunol.

[22] Ng LF, Chow A, Sun YJ, Kwek DJ, Lim PL (2009). IL-1beta, IL-6, and RANTES as biomarkers of Chikungunya severity.. PLoS One.

[23] Barksby HE, Lea SR, Preshaw PM, Taylor JJ (2007). The expanding family of interleukin-1 cytokines and their role in destructive inflammatory disorders.. Clin Exp Immunol.

[24] Tobler LH, Cameron MJ, Lanteri MC, Prince HE, Danesh A (2008). Interferon and interferon-induced chemokine expression is associated with control of acute viremia in West Nile virus-infected blood donors.. J Infect Dis.

[25] Pialoux G, Gauzere BA, Jaureguiberry S, Strobel M (2007). Chikungunya, an epidemic arbovirosis.. Lancet Infect Dis.

[26] Borgherini G, Poubeau P, Staikowsky F, Lory M, Le MN (2007). Outbreak of chikungunya on Reunion Island: early clinical and laboratory features in 157 adult patients.. Clin Infect Dis.

[27] Deshmane SL, Kremlev S, Amini S, Sawaya BE (2009). Monocyte chemoattractant protein-1 (MCP-1): an overview.. J Interferon Cytokine Res.

[28] Cronstein BN (2007). Interleukin-6--a key mediator of systemic and local symptoms in rheumatoid arthritis.. Bull NYU Hosp Jt Dis.

[29] Schaible HG, von Banchet GS, Boettger MK, Brauer R, Gajda M (2010). The role of proinflammatory cytokines in the generation and maintenance of joint pain.. Ann N Y Acad Sci.

[30] Domm S, Cinatl J, Mrowietz U (2008). The impact of treatment with tumour necrosis factor-alpha antagonists on the course of chronic viral infections: a review of the literature.. Br J Dermatol.

[31] Apte RN, Voronov E (2008). Is interleukin-1 a good or bad ‘guy’ in tumor immunobiology and immunotherapy?. Immunol Rev.

[32] de Jong MD, Simmons CP, Thanh TT, Hien VM, Smith GJ (2006). Fatal outcome of human influenza A (H5N1) is associated with high viral load and hypercytokinemia.. Nat Med.

[33] Spellberg B, Edwards JE (2001). Type 1/Type 2 immunity in infectious diseases.. Clin Infect Dis.

[34] Cameron MJ, Ran L, Xu L, Danesh A, Bermejo-Martin JF (2007). Interferon-mediated immunopathological events are associated with atypical innate and adaptive immune responses in patients with severe acute respiratory syndrome.. J Virol.

[35] Bermejo-Martin JF, Ortiz de LR, Pumarola T, Rello J, Almansa R (2009). Th1 and Th17 hypercytokinemia as early host response signature in severe pandemic influenza.. Crit Care.

[36] Bermejo-Martin JF, Garcia-Arevalo MC, Alonso A, De Lejarazu RO, Pino M (2007). Persistence of proinflammatory response after severe respiratory syncytial virus disease in children.. J Allergy Clin Immunol.

[37] Cameron CM, Cameron MJ, Bermejo-Martin JF, Ran L, Xu L (2008). Gene expression analysis of host innate immune responses during Lethal H5N1 infection in ferrets.. J Virol.

[38] Hanaoka R, Kasama T, Muramatsu M, Yajima N, Shiozawa F (2003). A novel mechanism for the regulation of IFN-gamma inducible protein-10 expression in rheumatoid arthritis.. Arthritis Res Ther.

[39] Ruschpler P, Lorenz P, Eichler W, Koczan D, Hanel C (2003). High CXCR3 expression in synovial mast cells associated with CXCL9 and CXCL10 expression in inflammatory synovial tissues of patients with rheumatoid arthritis.. Arthritis Res Ther.

[40] Ueno A, Yamamura M, Iwahashi M, Okamoto A, Aita T (2005). The production of CXCR3-agonistic chemokines by synovial fibroblasts from patients with rheumatoid arthritis.. Rheumatol Int.

[41] Aggarwal A, Agarwal S, Misra R (2007). Chemokine and chemokine receptor analysis reveals elevated interferon-inducible protein-10 (IP)-10/CXCL10 levels and increased number of CCR5+ and CXCR3+ CD4 T cells in synovial fluid of patients with enthesitis-related arthritis (ERA).. Clin Exp Immunol.

[42] Kuan WP, Tam LS, Wong CK, Ko FW, Li T (2010). CXCL 9 and CXCL 10 as Sensitive markers of disease activity in patients with rheumatoid arthritis.. J Rheumatol.

[43] Zeremski M, Markatou M, Brown QB, Dorante G, Cunningham-Rundles S (2007). Interferon gamma-inducible protein 10: a predictive marker of successful treatment response in hepatitis C virus/HIV-coinfected patients.. J Acquir Immune Defic Syndr.

[44] Nigrovic PA, Lee DM (2007). Synovial mast cells: role in acute and chronic arthritis.. Immunol Rev.

[45] Nigrovic PA, Lee DM (2005). Mast cells in inflammatory arthritis.. Arthritis Res Ther.

[46] Malbec O, Daeron M (2007). The mast cell IgG receptors and their roles in tissue inflammation.. Immunol Rev.

[47] Nandakumar KS, Holmdahl R (2006). Antibody-induced arthritis: disease mechanisms and genes involved at the effector phase of arthritis.. Arthritis Res Ther.

[48] Tsuboi N, Ernandez T, Li X, Nishi H, Cullere X (2011). Regulation of human neutrophil Fcgamma receptor IIa by C5a receptor promotes inflammatory arthritis in mice.. Arthritis Rheum.

